# The Importance of Natural Experiments in Diabetes Prevention and Control and the Need for Better Health Policy Research

**DOI:** 10.5888/pcd10.120145

**Published:** 2013-01-31

**Authors:** Edward W. Gregg, Mohammed K. Ali, Bernice A. Moore, Meda E. Pavkov, Heather M. Devlin, Sanford Garfield, Carol M. Mangione

**Affiliations:** Author Affiliations: Edward W. Gregg, Mohammed K. Ali, Bernice A. Moore, Heather M. Devlin, Centers for Disease Control and Prevention, Atlanta, Georgia; Sanford Garfield, National Institutes of Health, Bethesda, Maryland; Carol M. Mangione, University of California, Los Angeles, California.

Diabetes has steadily increased in prevalence, becoming one of the nation’s most challenging public health threats (1). Prevalence among adults is now more than 10%, and diabetes is the leading cause of nontraumatic lower-extremity amputation, end-stage kidney disease, and blindness; it more than doubles the risk of heart disease, stroke, and disability (1,2). Strong clinical trial evidence indicates that much of the illness caused by diabetes is preventable, further positioning diabetes as a public health priority (3,4) and stimulating a national emphasis on the quality of diabetes care and self-management (5–7). Although many such efforts have been successful, leading to better care, risk factor control, and reduced risk of complications, new challenges have arisen. The increases in obesity and in diabetes incidence demand that health systems and communities apply primary prevention strategies at the population level while simultaneously tackling the pervasive geographic and socioeconomic disparities in diabetes prevalence, care, and complications that remain (8,9).

Compared to the long list of clinical best practices to prevent diabetes complications, the evidence base is thin for population- and policy-level approaches to improve health behaviors, access to and delivery of care and preventive services, and the healthful attributes of communities. This imbalance of evidence calls for a new platform of public health research for diabetes. We contend that the imbalance can be corrected by a greater emphasis on natural experiments: rigorously designed quasi-experimental studies to investigate the health effects of naturally occurring population- and policy-level approaches emanating from health systems, communities, business organizations, and governments.

The gaps in evidence for naturally occurring population- and policy-level approaches have not resulted from a lack of such approaches. Numerous large-scale initiatives and health-related services to reduce the risk and consequences of diabetes are taking place. Employers, health plans, health systems, and communities regularly embark on screening and wellness programs and quality-improvement programs for entire populations; state and local governments have proposed or implemented policies such as taxes on unhealthful foods, vouchers for lifestyle and community programs, or restrictions on the way social services can be used. To remain competitive in a nation where large employers and government are the dominant purchasers of health insurance, health plans frequently develop new reimbursement and benefit designs that influence patterns of services provided to large populations. Finally, national and state legislatures adopt laws that fundamentally affect the access to and delivery, quality, and costs of care and preventive services for people at risk for or diagnosed with diabetes. By 2014, features of the Affordable Care Act of 2010 are likely to change access to services and quality of care, particularly for people who were previously uninsured.

The gaps in evidence for naturally occurring population- and policy-level approaches have resulted from a lack of rigorous health policy research: the objective, critical examination and evaluation of the benefits and drawbacks of such approaches. Health policy studies have typically lacked control conditions, which has limited the ability to distinguish between policy effects and secular trends and gauge true effectiveness (10). Randomized controlled trials establish causality and quantify efficacy under ideal conditions but are often impractical for the study of health policies in a complex world. Instead of seeking more rigorous nonrandomized alternatives, health policy research has frequently settled for cross-sectional or noncontrolled alternatives that lead to ambiguous or misleading conclusions.

Responding to both the need and opportunity for better health policy research for diabetes, the Centers for Disease Control and Prevention (CDC) and the National Institute of Diabetes and Digestive and Kidney Diseases has initiated a multicenter research network: Natural Experiments in Translation for Diabetes, or NEXT-D. The mission of NEXT-D is to examine the effectiveness of population-level health policies on diabetes prevention, control, and inequalities through rigorous health policy research. A collaborative approach was chosen because it facilitates multisite studies and the use of common measurements and indicators. Collaboration will also enhance the design, analysis, and dissemination of translational research. The ultimate goal of the collaboration is to provide stakeholders with a clear understanding of best practices that can be implemented by employers, health plans, health systems, communities, legislatures, or governments to prevent and control diabetes.

NEXT-D studies are also intended to inform the priorities of the CDC-funded Diabetes Prevention and Control Programs (DPCPs) in 58 state and territorial health departments (1). DPCPs bring together diverse stakeholders to implement population-based interventions to improve diabetes risk factors, control, and disparities and to drive state and territorial progress toward national public health objectives. Innovative DPCP strategies that are similar across states could become candidates for NEXT-D evaluations. Conversely, several components of the NEXT-D portfolio of natural experiments may have important implications for DPCPs, including diabetes care quality improvement, access to self-management education, access to lifestyle-based diabetes prevention programs, and healthy food environments in communities.

Articles in this *Preventing Chronic Disease* collection describe the NEXT-D natural experiments now under way — their rationale and importance, their design, and their intended effects. The objective of this collection is to share expertise and methods for addressing the complexity of real-world data. We hope to stimulate others to embark on and publish studies on natural experiments.

The NEXT-D studies have several attributes that will enhance their effect on diabetes health research and policies. First, interventions are being implemented naturally (ie, not for research purposes), and they take place among health systems, insurers, employers, the private sector, communities, and government agencies, each of which reaches a large population. As a result, study investigators do not use their own research funds for implementation, and interventions have high external generalizability. Second, the NEXT-D studies span several major public health themes, including the design of health care benefits, clinic–community partnerships, adoption of health information technology, and employer-based initiatives to screen and prevent diabetes. Third, the studies use longitudinal, controlled study designs involving diverse populations and rigorous analytic methods that aim to distinguish between policy effects and underlying trends. Fourth, through close partnerships with the organizations that implement these interventions in real-world settings, the NEXT-D studies will help to eliminate barriers to sustaining and disseminating approaches that are found to be effective at preventing and improving care for people who have diabetes. Fifth, by working in partnership with private sector and public policy decision makers, NEXT-D research teams can identify and analyze outcome indicators that are most informative (ie, provide actionable evidence) to those decision makers. Finally, the studies encompass primary and secondary prevention and complementary, nonredundant approaches. This new platform of public health research for diabetes — natural experiments — will fill the gaps in evidence for population- and policy-level approaches, correct the imbalance in the evidence base between clinical best practices and population- and policy-level approaches, and ultimately help to reduce the burden of diabetes.
