# Erratum, Vol. 10, January 31 Release

**DOI:** 10.5888/pcd10.120145e

**Published:** 2013-02-14

In the byline for “The Importance of Natural Experiments in Diabetes Prevention and Control and the Need for Better Health Policy Research,” the academic degree for Sanford Garfield, PhD, was corrected, and Meda Pavkov was changed to Meda E. Pavkov. The changes were made to our website on February 5, 2013, and appear online at www.cdc.gov/pcd/issues/2013/12_0145.htm.

